# Iatrogenic Femoral Arteriovenous Fistula After Cardiac Ablation Presenting As Heart Failure: Successful Endovascular Repair

**DOI:** 10.7759/cureus.111813

**Published:** 2026-06-30

**Authors:** Miguel A Peraza-Arjona, Victor M Ayuso-Diaz, Alfonso Peraza-Fernandez, Mayra A. Guachun-Guachun, Carla Munguia-Aguilar

**Affiliations:** 1 Angiology, Vascular and Endovascular Surgery, Regional Hospital “Elvia Carrillo Puerto” ISSSTE, Yucatán, MEX; 2 Research and Education, Medical Care and Research, Yucatan, MEX; 3 Genomic-Metabolic Unit, University Marista of Merida, Yucatan, MEX; 4 Research and Education, Clínica Yucatán, Yucatan, MEX; 5 Angiology, Vascular and Endovascular Surgery, Regional Hospital "Elvia Carrillo Puerto" ISSSTE, Yucatán, MEX; 6 Angiology, Vascular and Endovascular Surgery, Occidental General Hospital "Zoquipan", Jalisco, MEX; 7 Angiology, Vascular and Endovascular Surgery, High Specialty Hospital "Centennial of the Mexican Revolution" ISSSTE, Morelos, MEX

**Keywords:** arteriovenous fistula, cardiac ablation, common femoral artery, covered stent, endovascular repair, femoral artery, femoral vein, high-flow fistula, iatrogenic complication, vascular access

## Abstract

Iatrogenic femoral arteriovenous fistulas (AVFs) are an uncommon but potentially significant vascular complication following catheter-based cardiac procedures, particularly those requiring femoral access. Although most AVFs remain small and hemodynamically insignificant, high-flow fistulas may result in substantial arteriovenous shunting, leading to increased cardiac preload, reduced systemic vascular resistance, and high-output heart failure. Early recognition is essential to prevent progressive hemodynamic deterioration and cardiovascular compromise.

The clinical presentation of femoral AVFs is variable and may range from asymptomatic findings to overt heart failure. Imaging plays a central role in diagnosis and therapeutic planning, with computed tomography angiography enabling anatomical characterization and angiography allowing definitive diagnosis and simultaneous intervention.

We report a case of symptomatic high-flow femoral AVF after cardiac ablation successfully treated with endovascular covered stent placement. Endovascular repair achieved complete exclusion of the fistulous communication with restoration of normal arterial flow and a favorable clinical outcome.

During follow-up, the patient remained asymptomatic, with sustained stent patency and no evidence of recurrent arteriovenous flow. This case highlights the importance of maintaining a high index of suspicion for vascular access-related complications in patients presenting with unexplained heart failure after catheter-based procedures. It also supports the role of endovascular repair as a safe, effective, and minimally invasive treatment strategy for symptomatic high-flow femoral AVFs.

## Introduction

Vascular access is an essential component of catheter-based cardiac ablation procedures, which are increasingly performed for the management of atrial and ventricular arrhythmias. The widespread use of femoral access, often requiring multiple venous and occasionally arterial punctures, has led to a growing recognition of access-site complications despite advances in technique and device technology [1,2]. Although most complications are minor, including hematoma or prolonged hemostasis time, clinically significant vascular injuries continue to occur and remain a relevant source of morbidity. [1]

Among these, iatrogenic femoral arteriovenous fistulas (AVFs) represent an uncommon but potentially serious complication. The reported incidence of major femoral vascular complications following catheter ablation ranges between 0.2% and 1.5%, with AVFs constituting a small proportion of these events [1]. Iatrogenic vascular injuries, including AVFs, pseudoaneurysms, and thrombosis, are primarily associated with percutaneous vascular cannulation, particularly at the femoral level, which remains the most frequently accessed site [3]. While many AVFs are small and may resolve spontaneously, their clinical significance depends largely on the magnitude of the arteriovenous shunt.

The pathophysiological impact of an AVF is driven by the direct communication between the high-pressure arterial system and the low-resistance venous circulation, resulting in increased venous return, reduced systemic vascular resistance, and a compensatory rise in cardiac output. Over time, this persistent volume overload may lead to ventricular dilation, increased cardiac workload, and ultimately high-output heart failure, a condition that remains under-recognized but carries significant morbidity and mortality if untreated [4]. Clinically, patients may present with progressive dyspnea, signs of heart failure, localized pain, or a continuous bruit at the access site, reflecting the hemodynamic burden imposed by the fistulous communication [5].

Despite its clinical relevance, there is currently no standardized guideline for the management of iatrogenic femoral AVFs. Therapeutic strategies are largely individualized and depend on symptom severity, shunt flow, and hemodynamic consequences. While conservative management may be appropriate for small, asymptomatic fistulas, intervention becomes necessary in cases of symptomatic or high-flow lesions [3]. Historically, surgical repair was considered the definitive treatment; however, advances in endovascular techniques, including stent graft placement and embolization, have shifted the paradigm toward minimally invasive approaches, offering high success rates with reduced procedural morbidity [6,7].

In this context, we present a case of a symptomatic high-flow iatrogenic femoral AVF following cardiac ablation, successfully managed with endovascular covered stent placement. This report highlights the importance of early recognition, appropriate diagnostic evaluation, and individualized therapeutic decision-making in the management of this rare but clinically significant complication.

## Case presentation

A 58-year-old woman with a history of type 2 diabetes mellitus, dyslipidemia, hypothyroidism, severe pulmonary hypertension, and atrial fibrillation with prolonged pauses had recently undergone percutaneous pulmonary vein isolation and cavotricuspid isthmus ablation. One month later, she required a repeat intervention consisting of cryoablation of the same isthmus, both procedures performed via left femoral venous access.

Within one week following the most recent procedure, the patient developed progressively worsening exertional dyspnea, orthopnea, marked fatigue, and bilateral lower extremity edema. Her symptoms evolved rapidly, leading to significant functional limitation, without prior recent episodes of cardiac decompensation.

On physical examination, she was found to have jugular venous distension, bibasilar crackles, and bilateral pitting edema extending to the mid-tibial region. Localized tenderness was noted at the left inguinal region corresponding to the vascular access site, where a continuous bruit was auscultated throughout the cardiac cycle. Distal pulses were preserved, with no signs of peripheral ischemia.

Given the suspicion of a vascular access-related complication, computed tomography angiography of the lower extremities was performed. Imaging revealed an arteriovenous communication between the common femoral artery and the superficial femoral artery draining into the femoral vein, consistent with a high-flow arteriovenous fistula with early venous opacification (Figure 1). 

**Figure 1:**
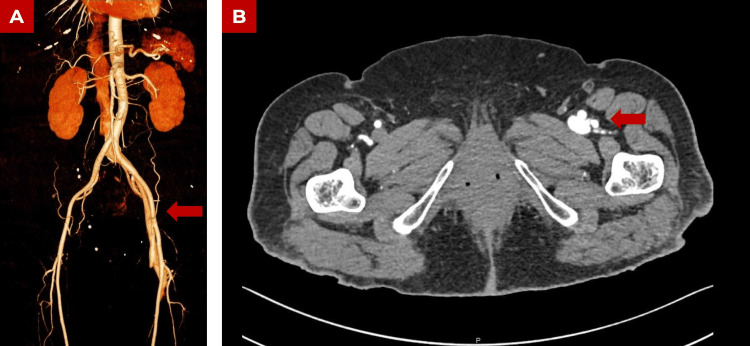
Computed tomography angiography demonstrating a high-flow femoral arteriovenous fistula A) Three-dimensional reconstruction showing abnormal communication at the level of the left femoral bifurcation, suggestive of an arteriovenous fistula (arrow). (B) Axial computed tomography image demonstrating early opacification of the femoral vein adjacent to the femoral artery, consistent with a high-flow arteriovenous shunt (arrow).

In the context of symptomatic high-output physiology secondary to the arteriovenous shunt, an endovascular approach was selected.

A retrograde approach was performed via distal puncture of the left superficial femoral artery at the level of Hunter’s canal. Diagnostic arteriography confirmed a significant arteriovenous fistula at the femoral bifurcation, with immediate contrast passage into the deep venous system. Detailed angiographic measurements of the involved segments were obtained to guide appropriate device selection (Figure 2).

**Figure 2:**
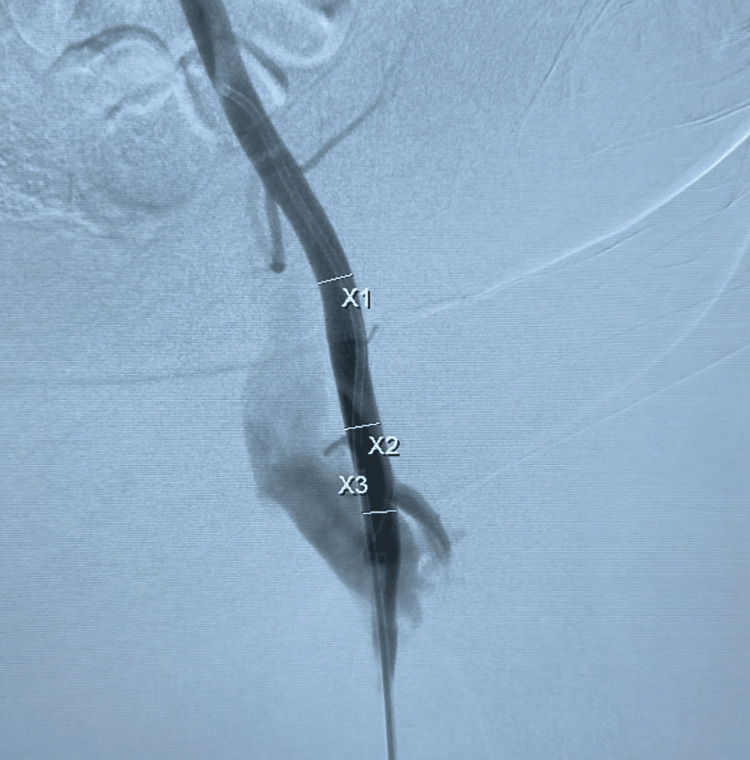
Diagnostic angiography with morphological characterization and measurements for endovascular planning Angiography of the left lower extremity demonstrating a high-flow femoral arteriovenous fistula at the level of the femoral bifurcation, with early opacification of the deep venous system. Angiographic measurements of the involved arterial segments (X1 = 6.47 mm, X2 = 6.81 mm, X3 = 6.30 mm) are shown, which were essential for accurate selection of the diameter and length of the covered stents used for endovascular exclusion of the fistulous communication.

Two polytetrafluoroethylene-covered stents (7 × 37 mm and 7 × 57 mm) were successfully deployed, achieving complete exclusion of the fistulous communication. Final angiography demonstrated restoration of normal arterial flow, adequate distal perfusion, and no evidence of residual leakage (Figure 3).

**Figure 3:**
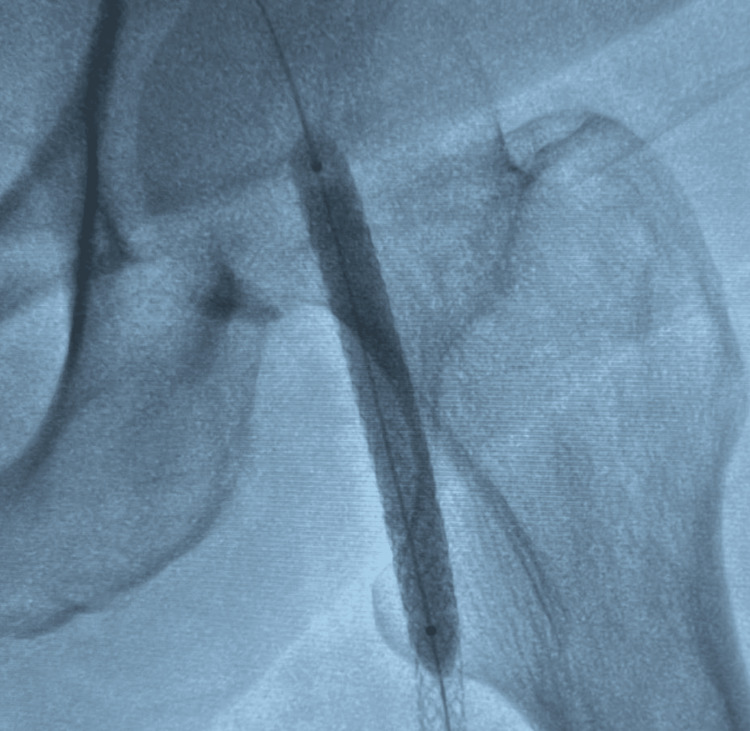
Fluoroscopic image showing deployment of covered stent for endovascular exclusion of femoral arteriovenous fistula Fluoroscopic image demonstrating deployment of a polytetrafluoroethylene-covered stent at the level of the left femoral bifurcation during endovascular treatment of the arteriovenous fistula. The device shows appropriate expansion and accurate positioning across the fistulous segment prior to final angiographic confirmation.

The patient exhibited rapid clinical improvement following the procedure, with resolution of dyspnea and progressive reduction of peripheral edema. Immediate post-procedural angiographic control confirmed complete exclusion of the arteriovenous communication, with restoration of normal arterial flow and no evidence of contrast extravasation (Figure 4). 

**Figure 4:**
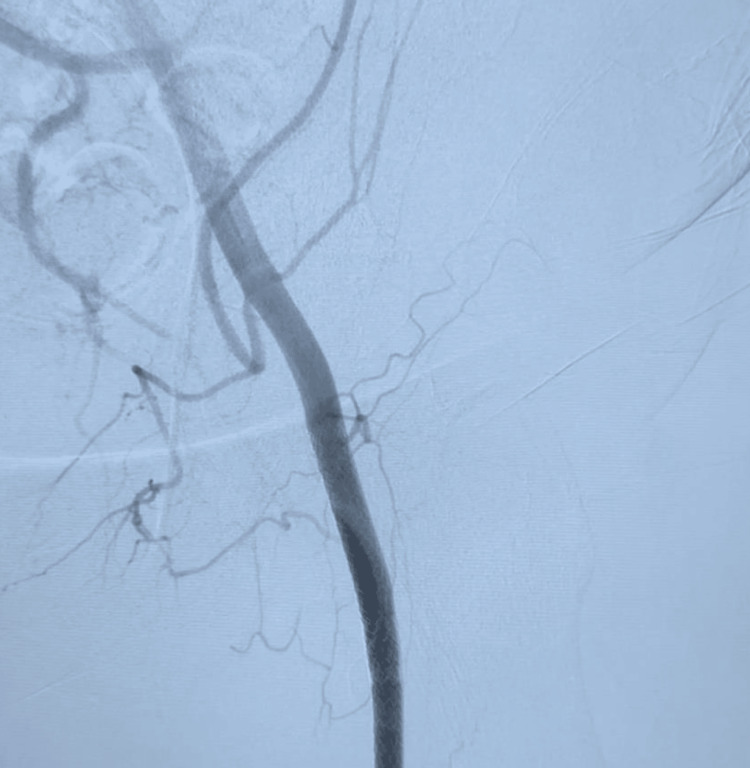
Final angiographic control demonstrating complete exclusion of femoral arteriovenous fistula Final angiographic image showing successful exclusion of the arteriovenous fistula following covered stent deployment at the femoral bifurcation. Normal antegrade arterial flow is restored, with no evidence of early venous opacification, residual shunting, or contrast extravasation.

At follow-up visits at 1, 3, and 6 months, the patient remained asymptomatic, with no clinical signs of heart failure. Follow-up computed tomography angiography (CTA) demonstrated sustained stent patency and absence of residual or recurrent arteriovenous flow (Figure 5).

**Figure 5:**
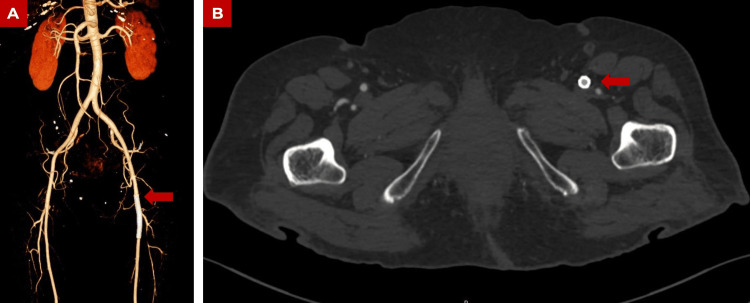
Follow-up computed tomography angiography confirming durable exclusion of femoral arteriovenous fistula (A) Three-dimensional computed tomography angiography reconstruction demonstrating preserved arterial continuity at the left femoral bifurcation without evidence of abnormal arteriovenous communication (arrow). (B) Axial computed tomography image showing absence of early venous opacification adjacent to the femoral vessels, confirming sustained exclusion of the fistula and no residual or recurrent shunting (arrow).

This case illustrates the evolution of an initially subclinical iatrogenic femoral arteriovenous fistula into a clinically significant high-output state resulting in new-onset heart failure. Endovascular exclusion using covered stents provided a safe and effective treatment, achieving durable closure of the fistula while avoiding open surgical intervention and preserving contralateral vascular access for potential future procedures. 

## Discussion

Vascular access-site complications remain an inherent risk of catheter-based diagnostic and therapeutic procedures, particularly when femoral access is employed. The increasing use of percutaneous interventions has been paralleled by a rise in vascular complications, with reported incidences ranging from 0.2% to 1%, and higher rates observed in therapeutic procedures due to larger device calibers and the use of anticoagulation and antiplatelet therapy [8]. Among these complications, iatrogenic arteriovenous fistulas (AVFs) represent an uncommon but clinically relevant entity.

The pathophysiology of iatrogenic AVFs is primarily related to inadvertent simultaneous arterial and venous puncture during vascular access, resulting in a persistent arteriovenous communication. While many AVFs remain small and clinically silent, high-flow fistulas can induce significant hemodynamic alterations. These include reduced systemic vascular resistance, increased venous return, and compensatory elevation in cardiac output, ultimately leading to a high-output state. In advanced cases, chronic volume overload may result in ventricular dilatation, pulmonary hypertension, and right heart remodeling. Importantly, delayed closure of AVFs has been associated with incomplete reverse cardiac remodeling, emphasizing the importance of early diagnosis and timely intervention [9].

Clinically, the presentation of femoral AVFs varies widely, ranging from asymptomatic findings to overt heart failure. Physical examination remains a critical component of diagnosis, with a continuous bruit at the access site serving as a key finding. In our case, the combination of new-onset heart failure symptoms and a continuous inguinal murmur following femoral access strongly suggested a hemodynamically significant AVF, consistent with previously reported cases of high-output heart failure secondary to arteriovenous shunting [9].

Risk factors for vascular access complications have been increasingly studied. Procedural factors such as prolonged intervention time, repeated punctures, and complexity of the intervention have been identified as significant predictors of complications [10,11]. Additionally, patient-related factors, including comorbidities such as diabetes mellitus, hypertension, and vascular disease, further increase susceptibility to vascular injury. These elements likely contributed to the development of the AVF in our patient, who underwent multiple femoral access procedures.

Imaging plays a pivotal role in both diagnosis and therapeutic planning. Computed tomography angiography (CTA) enables detailed anatomical characterization of the fistulous communication and identification of early venous opacification, whereas diagnostic angiography remains the gold standard for definitive assessment and simultaneous endovascular intervention. In the present case, angiographic measurements were essential for accurate device sizing and procedural planning, allowing complete exclusion of the fistula while preserving adequate distal arterial perfusion.

Management of iatrogenic femoral AVFs is not standardized and must be individualized based on symptomatology and hemodynamic impact. Conservative management may be appropriate for small, asymptomatic fistulas; however, intervention is warranted in symptomatic or high-flow lesions. Historically, surgical repair was considered the gold standard, achieving high success rates but at the expense of increased morbidity, longer recovery times, and complication rates reported up to 25% in some series [8].

In recent years, endovascular approaches have emerged as the preferred treatment modality. Percutaneous techniques offer a minimally invasive alternative with high technical success rates, reduced hospital stay, and lower morbidity. Large series have demonstrated technical success rates exceeding 90% in the percutaneous treatment of vascular access complications, supporting their role as first-line therapy [8]. The use of polytetrafluoroethylene (PTFE)-covered stents allows immediate exclusion of the fistulous communication while maintaining arterial patency.

Long-term outcomes of endovascular repair are also favorable. Studies evaluating PTFE-covered stent-grafts have reported primary patency rates of approximately 77% at three years, with even higher patency rates observed in AVFs compared to other vascular pathologies. These findings support the durability and effectiveness of endovascular repair in appropriately selected patients. Furthermore, contemporary vascular practice has shifted toward an endovascular-first strategy, reserving open surgery for complex or anatomically unsuitable cases [12].

Comparative analyses between open and endovascular repair provide additional context. While open repair may offer lower reintervention rates in certain scenarios, endovascular techniques provide advantages in terms of reduced invasiveness, shorter recovery time, and lower perioperative risk, particularly in patients with significant comorbidities [13]. These characteristics make endovascular therapy especially suitable in cases requiring rapid hemodynamic stabilization, such as high-flow AVFs.

In the present case, endovascular treatment with PTFE-covered stents resulted in complete and durable exclusion of the AVF, with rapid clinical improvement and sustained symptom resolution during follow-up. This outcome is consistent with current evidence supporting the safety and efficacy of endovascular approaches in the management of iatrogenic femoral AVFs [14].

This case underscores several important clinical implications. First, clinicians should maintain a high index of suspicion for vascular access complications in patients presenting with unexplained heart failure following catheter-based procedures. Second, early imaging evaluation is essential to establish the diagnosis and guide management. Finally, endovascular intervention represents a safe, effective, and minimally invasive therapeutic strategy that provides excellent immediate and long-term outcomes, supporting its role as the preferred treatment modality in most cases.

## Conclusions

Iatrogenic femoral arteriovenous fistula is an uncommon but potentially significant complication of femoral vascular access in catheter-based procedures. Although frequently asymptomatic, high-flow fistulas may lead to clinically relevant hemodynamic alterations, including high-output heart failure. Early recognition based on clinical suspicion and appropriate imaging is essential to prevent progression and guide timely management.

Endovascular treatment using covered stents represents a safe, effective, and minimally invasive therapeutic option, allowing immediate exclusion of the fistulous communication while preserving arterial patency. This case underscores the importance of considering vascular access-related complications in patients with new-onset heart failure after cardiac interventions and supports the use of endovascular approaches as a first-line strategy in appropriately selected cases.

## References

[1] Ishikawa E, Miyazaki S, Mukai M (2020). Femoral vascular complications after catheter ablation in the current era: the utility of computed tomography imaging. J Cardiovasc Electrophysiol.

[2] Gupta S, Kolluri R, Simoes T (2024). Safety of multi-access site venous closure following catheter ablation of atrial fibrillation and flutter. J Interv Card Electrophysiol.

[3] Son J, Kim J, Choi KH (2026). High-output heart failure associated with arteriovenous fistula. JACC Case Rep.

[4] Volk MC, Honnekeri B, Ghobrial J (2025). High-output heart failure from arteriovenous dialysis access: a structured approach to diagnosis and management. Cleve Clin J Med.

[5] Tobe A, Tanaka A, Yoshida S (2021). High-output heart failure caused by a tumor-related arteriovenous fistula: a case report and literature review. Intern Med.

[6] Reig AS, Simon SD, Neblett WW 3rd, Mericle RA (2010). Eight-year follow-up after palliative embolization of a neonatal intracranial dural arteriovenous fistula with high-output heart failure: management strategies for symptomatic fistula growth and bilateral femoral occlusions in pediatric patients. J Neurosurg Pediatr.

[7] Chikata A, Kato T, Usuda K (2024). Preclose versus postclose using suture-mediated vascular closure system for catheter ablation with femoral vein access. JACC Clin Electrophysiol.

[8] Minici R, Paone S, Talarico M, Zappia L, Abdalla K, Petullà M, Laganà D (2020). Percutaneous treatment of vascular access-site complications: a ten years' experience in two centres. CVIR Endovasc.

[9] He T, He X, Yuan XM (2025). High-output heart failure secondary to iatrogenic arteriovenous fistula: a case report. World J Cardiol.

[10] Gopar-Nieto R, Huerta-Liceaga F, Chávez-Gómez NL (2019). Risk factors and temporal trends for vascular access-related complications in coronary procedures: evolving from femoral to radial approach. Arch Cardiol Mex.

[11] Calotta NA, Astor BC, Ross JR (2023). Automated 3D ultrasound enables novice users to measure arteriovenous fistula maturation parameters with comparable accuracy to conventional duplex by trained sonographers: results of a benchtop study. J Vasc Access.

[12] Topcuoglu OM, Altay CM, Alpar A, Kucukay F (2020). Predictors of long-term outcomes after polytetrafluoroethylene-covered stent-graft repair of peripheral arterial aneurysms, pseudo-aneurysms, and arterio-venous fistulas. Vascular.

[13] Yan Q, Davies MG (2024). Comparison of open and endovascular aneurysm repair in native arteriovenous fistulas. Ann Vasc Surg.

[14] Miceli F, Demirxhiu G, Di Girolamo A (2025). Mid- and long-term results of covered stents for iatrogenic common femoral artery injury. Biomedicines.

